# White matter and impulsivity are prospectively associated with non‑suicidal self‑injury in youth: evidence for sex differences

**DOI:** 10.1038/s41386-026-02458-3

**Published:** 2026-06-08

**Authors:** Mindy Westlund Schreiner, Sarah J. Manuele, Jason J. Washburn, Stew Shankman, Kathryn R. Cullen, Lei Wang, Lisanne M. Jenkins

**Affiliations:** 1 Behavioral Health, Nationwide Children’s Hospital, Columbus, OH USA; 2 Department of Psychiatry and Behavioral Health, Ohio State University, Columbus, OH USA; 3 Department of Psychiatry, School of Translational Medicine, Monash University, Melbourne, VIC Australia; 4 Department of Psychiatry and Behavioral Sciences, Feinberg School of Medicine, Northwestern University, Chicago, IL USA; 5 Stephen M. Stahl Center for Psychiatric Neuroscience, Department of Psychiatry and Behavioral Sciences, Northwestern University, Chicago, IL, USA; 6 Department of Psychiatry and Behavioral Sciences, University of Minnesota, Minneapolis, MN USA; 7 Rotman Research Institute, Baycrest Academy for Research and Education, Toronto, ON Canada; 8 Department of Psychiatry, School of Medicine, The University of Melbourne, Melbourne, VIC Australia

**Keywords:** Human behaviour, Neuronal development

## Abstract

Non-suicidal self-injury (NSSI) typically begins in adolescence and is associated with higher impulsivity and compromised white matter microstructure in the brain. Identifying early predictors of NSSI is a high priority. We used data from the Adolescent Brain Cognitive Development Study to examine whether baseline white matter, specifically generalized fractional anisotropy (GFA), and different facets of impulsivity were prospectively associated with NSSI onset two years later (Y_2_). We also examined interactions with sex. We identified 209 youth aged 8–11 years who reported NSSI at Y_2_, but not at baseline (incident NSSI; iNSSI) and 209 controls matched by demographic and clinical characteristics. We conducted a Least Absolute Shrinkage and Selection Operator (LASSO) regularization, which yielded an AUC of .60, to narrow down predictors for logistic regression. Significant main effects indicated that lower GFA of the left parolfactory section of the cingulum (OR = 0.63) and higher negative urgency (OR = 1.54) were prospectively associated with Y_2_ NSSI. Sex interactions indicated that the relations between Y_2_ NSSI and lower GFA of the left parolfactory section of the cingulum (OR = 1.75) and higher negative urgency (OR = 0.60) were driven by males. Higher sensation seeking (OR = 1.36) and lack of perseverance (OR = 1.46) were more strongly associated with Y_2_ NSSI among females than males. Future work is needed to better understand the course of development of white matter microstructure and impulsivity as it relates to NSSI.

## Introduction

Non-suicidal self-injury (NSSI), or the act of deliberately harming one’s own body tissue without the intention of death, is a growing public health concern [1]. A recent estimate of the prevalence of NSSI among youth is around 22% [2] with onset typically occurring between 12 and 14 years of age [3]. The most common function of NSSI is emotion regulation, particularly reducing negative affect [4–7]. Brain regions important for emotion regulation, including frontolimbic regions, often show aberrant functioning in individuals with NSSI [8]. Further, impulsivity is elevated among individuals with NSSI and a recent study found that combining self-reported measures of emotion regulation and impulsivity yielded greater accuracy in discriminating youth with versus without NSSI [9]. Specific facets of impulsivity that have been associated with NSSI include negative and positive urgency (the tendency to act rashly in the context of heightened emotions), lack of premeditation and perseverance, and sensation seeking [10, 11]. While these cross-sectional studies are limited in capturing whether impulsivity precedes NSSI onset, one meta-analysis of longitudinal studies found that higher baseline impulsivity was associated with increased NSSI risk among adolescents [12].

NSSI is also associated with alterations in neurobiological structure and function relative to controls. Neuroimaging studies have found that individuals with NSSI demonstrate disrupted neural connectivity and activation at rest and during task paradigms involving social rejection, cognitive control, and processing of emotions, threat, and pain [8, 13–16]. Fewer studies have examined structural connectivity associated with NSSI. Specifically, studies using diffusion weighted imaging (DWI) provide insight into the white matter organization within the brain. In the context of typical neurodevelopment, white matter volume and density increases from childhood through adolescence [17], with lower organization of global white matter microstructure associated with more general psychopathology in children [18]. As adolescence is a particularly vulnerable period for brain development, including that of white matter, understanding the relation between white matter organization and NSSI can provide important insights into how we can foster resilience in neurodevelopment to improve functional outcomes [19–24].

Extant research examining DWI metrics associated with NSSI are cross-sectional and none include enough male participants to examine sex effects. Relative to non-clinical controls, individuals with NSSI have demonstrated evidence of reduced white matter organization in several white matter tracts including the uncinate fasciculus (UF) and cingulum [25–28]. These regions are of particular interest in NSSI given their relation to impulsivity, which is strongly implicated in NSSI using self-report [10]. The UF serves as a structural connection between frontolimbic regions and has been implicated in emotion dysregulation, impulsivity, and borderline personality disorder (BPD) [29–32]. In NSSI, lower white matter organization of the UF is associated with impulsivity [26]. Further, the UF is one of the last white matter tracts to reach maturity [33, 34], potentially being a key target for intervention and of particular utility when examining early adolescence, as it may be less susceptible to heterogeneity due to age and pubertal status. The cingulum is also implicated in impulsivity due to its involvement in cognitive control [35], including attention and inhibitory control in children [36]. Specifically, cingulum white matter organization is associated with facets of impulsivity including sensation seeking in males and females, urgency in males, and lack of premeditation in females [37]. Lower measures of white matter organization are also associated with longer duration of engaging in NSSI, making it of particular interest to examine longitudinally prior to NSSI onset [26]. Importantly, the cingulum is large tract that likely serves a wide range of functions including emotion regulation and affective experiences, cognitive control, and inhibition [35]. Continued work must examine segments of the cingulum as opposed to a unitary tract.

Theoretical models of NSSI emphasize the role of emotion dysregulation as a proximal mechanism in the pathway to NSSI, with neurobiological vulnerabilities increasingly recognized as distal risk factors that may influence these processes over the course of development [38]. Within this framework, white matter organization of tracts supporting emotion regulation and impulsivity, such as the UF and cingulum, represent plausible neurobiological underpinnings that may be present prior to onset of NSSI. The present study builds upon prior research by leveraging neuroimaging and clinical data from the Adolescent Brain Cognitive Development^SM^ Study (ABCD Study®). The ABCD Study includes a large sample of adolescents with data collected at multiple time points, allowing for longitudinal analyses that can examine characteristics that are prospectively associated with NSSI onset as well as sex differences. Here, we examine how the organization of key white matter tracts (the UF and cingulum) and a self-reported measure of impulsivity at baseline (Y_0_) are associated with the onset of NSSI by Year 2 (Y_2_). Given prior research, we anticipate that youth with lower organization of the UF and cingulum at Y_0_ and higher levels of impulsivity, particularly negative urgency, will be more likely to report onset of NSSI at Y_2_. Consistent with the grant funding this work (R01MH132920), we hypothesize that more anterior segments of the cingulum, such as the parolfactory and frontal-parietal segments, would be prospectively associated with NSSI given that these segments serve more brain regions involved in inhibitory control. Further, we also examine the interaction of these predictors with sex, as the neurobiological underpinnings of NSSI in males have been particularly underexamined due to prior studies either excluding or having few males in their sample.

## Method

### Participants

Participants were selected from the ABCD Study (data release 5.1), a multisite longitudinal study that includes 11,868 youth aged 8–11 years (Y_0_) and their caregivers. Further details about the ABCD Study are described elsewhere [39]. For the present study, participants were youths who completed MRI scanning at baseline and self-reported NSSI occurring between Y_0_ and Y_2_ at the Y_2_ assessment (incident NSSI group; iNSSI). The iNSSI participants were then matched with controls using the procedure detailed below. Participants from the iNSSI or control groups were excluded if they reported current or lifetime self-injurious thoughts and behaviors (including NSSI) at Y_0_. The ABCD study was approved by the Institutional Review Boards at the local sites and caregivers and children provided written informed consent and assent, respectively. For the purposes of the present study, the IRB at Nationwide Children’s Hospital determined this project to be non-human subjects research.

#### Matching procedure

We developed a custom script in MATLAB to match each iNSSI participant with a control based on several demographic variables: adoption status, sex, gender identity, race, ethnicity, parental marital status, parental combined income, and pubertal status (parent and youth reported). Further, participants were also matched on current or past bipolar disorder (including whether current or most recent episode was manic, depressed, or hypomanic), major or unspecified depressive disorder (including remission status), disruptive mood dysregulation disorder, social anxiety disorder, and generalized anxiety disorder. Of the 209 iNSSI participants, 153 matched to a control on all variables. We then matched on all variables except for parent-reported pubertal status and matched an additional 11 iNSSI participants. We then removed race, ethnicity, and parental marital status, and re-added parental-reported puberty variables and 41 participants matched to a control. After also removing combined parental income, two iNSSI participants matched to a control. One remaining iNSSI participant was matched after removing diagnostic matching variables. The final iNSSI participant was matched to a hand-selected control based on age, sex, and non-binary gender identity.

### Measures

#### Clinical assessment

Presence of self-injurious thoughts and behaviors (including NSSI) in addition to other mental health symptoms and mood disorder diagnosis was determined using the Kiddie Schedule for Affective Disorders and Schizophrenia for DSM-5 (KSADS-5) [40], which was administered to youths and their caregivers separately at Y_0_ and Y_2_. The KSADS-5 youth interview at Y_2_ was used to determine NSSI onset_._ Specifically, a threshold rating for NSSI in the KSADS-5 is defined as “Has engaged in the behavior more than 5 times and/or has engaged in the behavior with significant injury to self (e.g., burn left scar, cut required stitches)”. Impulsivity was measured using the abbreviated youth self-report version of the Urgency, Premeditation, Perseverance, Sensation Seeking, Positive Urgency, Impulsive Behavior (UPPS-P) [41], completed by the youth at Y_0_.

#### Neuroimaging acquisition and analysis

ABCD data were acquired across 21 sites using scanners from three manufactures (Siemens, Philips, and GE). However, the present study did not include participants who were scanned using Philips scanners due to differences in DWI acquisition parameters. For Siemens and GE scanners, DWI data were acquired with a single acquisition using seven b = 0 volumes and four b-values of 500 (6 directions), 1000 (15 directions), 2000 (15 directions), and 3000 (60 directions). Slice thickness was 1.7 mm and data were rotated to align with the AC-PC at an isotropic resolution of 1.7 mm. DWI reconstruction used generalized q-sampling imaging [42] with a diffusion sampling length ratio of 1.25. Tensor metrics were calculated with b-value lower than 1750 s/mm^2^.

**Fig. 1:**
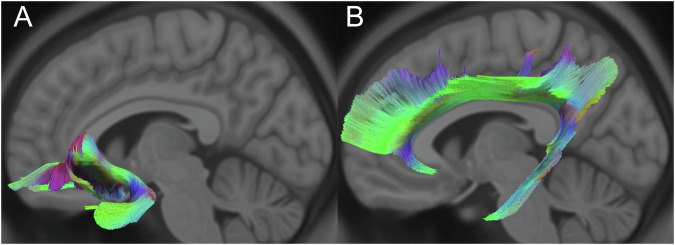
Uncinate fasciculus and cingulum tracts. Tracts shown above were generated using DSI studio. Generalized fractional anisotropy was extracted from these regions: **A** uncinate fasciculus and **B** cingulum.

**Fig. 2:**
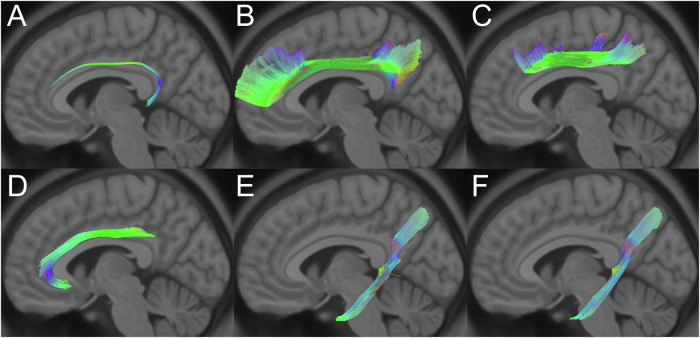
Sub-regions of the cingulum. The cingulum tract was subdivided into six sections: **A** frontal-parahippocampal, **B** frontal-parietal, **C** superior longitudinal fasciculus, **D** parolfactory, **E** parahippocampal, **F** parahippocampal-parietal.

DWI provides information about white matter microstructure via metrics such as fractional anisotropy (FA) or generalized fractional anisotropy (GFA), which provide information about the directionality of water diffusion, with GFA better able to resolve issues of multiple fiber directions in a voxel [43–45]. To align with prior research [26], we extracted GFA values from the right and left UF and cingulum (Fig. 1). However, we differed from prior work by using DSI Studio [46] to extract GFA values as opposed to custom-build tools in MATLAB. Further, rather than only examining the cingulum in its entirety, DSI Studio provided GFA of six (12 bilateral) regions of the cingulum: overall, frontal-parahippocampal, frontal-parietal, parahippocampal-parietal, parahippocampal, and parolfactory (Fig. 2). White matter tracts were mapped by nonlinearly warping a population-based tractography atlas to the native space [47].

### Statistical analyses

After extracting GFA values created by DSI Studio, we used R to complete analyses [48]. Predictors included 12 cingulum GFA, 2 UF GFA, 5 subscales from the UPPS-P, MRI manufacturer, and intracranial volume. All continuous predictors were converted to z-scores to aid in interpretability. We included sex × predictor interaction terms for all predictors, yielding a full design matrix that included both main effects and their interactions with sex. Our approach included using least absolute shrinkage and selection operator (LASSO) regularization to reduce dimensionality. This was followed by mixed-effects logistic regression that led to interpretable effect estimates while accounting for clustering due to site effects. We conducted LASSO regularization using the *glmnet* package in R within the *caret* framework. We used a standard LASSO with alpha = 1. A grid of 20 lambda values ranging from 0.001 to 0.1 was evaluated, with the optimal lambda selected based on maximum cross-validated AUC. The model was trained using repeated cross-validation (10-fold, repeated 5 times) to optimize the area under the receiver operating characteristic curve (AUC). Cross-validation folds were constructed using site-grouped *k-*fold partitioning to prevent data leakage from the multi-site structure of the dataset and ensure that all observations from a given site appeared in only one fold. Data collection site was not included as a predictor in the LASSO but was used to define grouped cross-validation folds and was included as a random intercept in the final mixed-effects model. We then fit a final LASSO model on the full dataset using the optimal lambda. Predictors with non-zero coefficients were entered as fixed effects into a mixed-effects logistic regression model using *glmer* from the *lme4* package, with a random intercept for site to account for variability between data collection sites. We fit the model using the BOBYQA optimizer with a maximum of 200,000 function evaluations.

## Results

### Participants

**Fig. 3:**
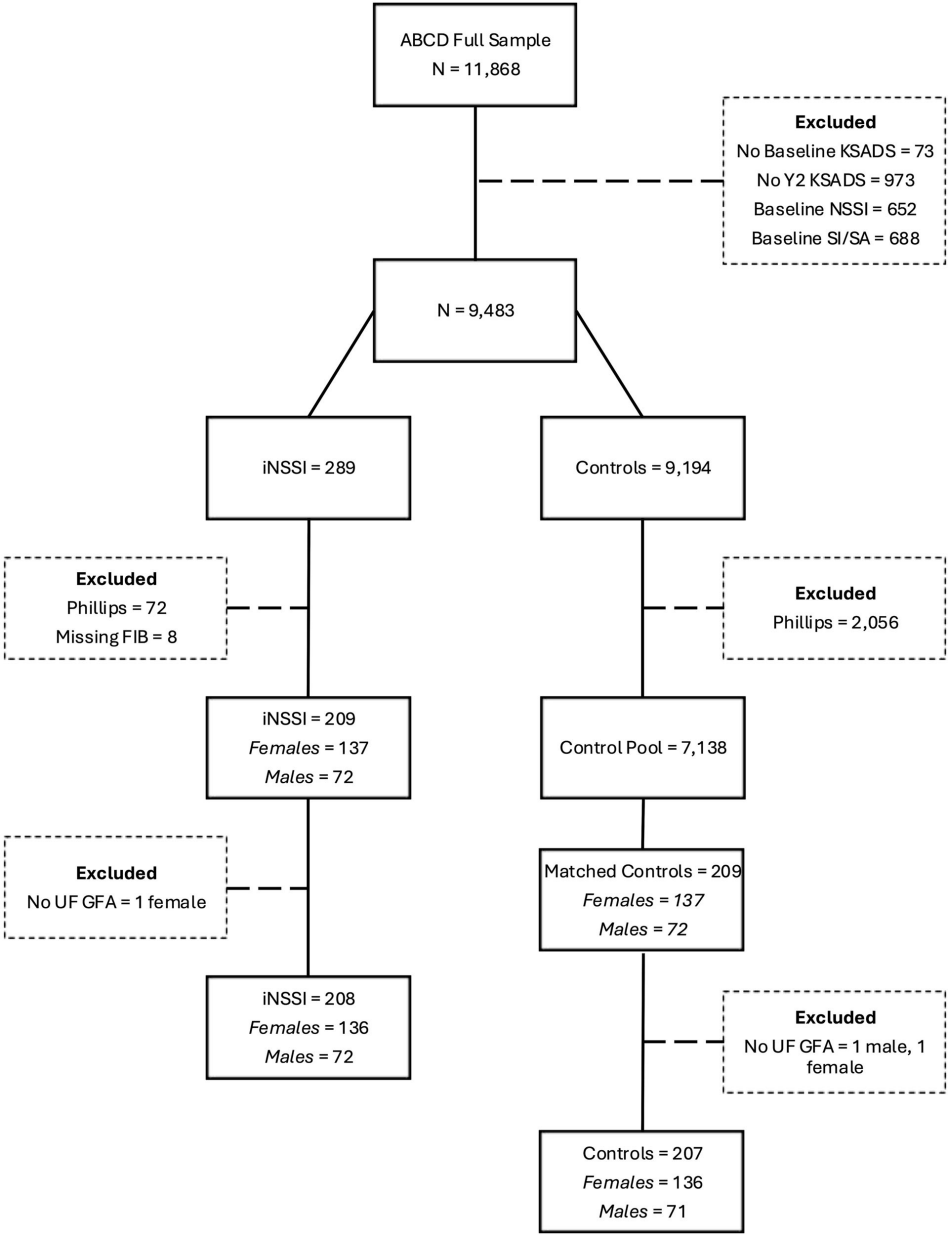
Participant flow diagram. ABCD Adolescent Brain Cognitive Development, KSADS Kiddie Schedule for Affective Disorders and Schizophrenia, NSSI Non-Suicidal Self-Injury, SI/SA Suicidal Ideation/Suicide Attempt, iNSSI Incident NSSI, FIB file type containing fiber direction and anisotropy information, UF Uncinate Fasciculus, GFA Generalized Fractional Anisotropy.

A total of 209 youths with NSSI (137 female and 72 male) and 209 matched controls had DWI data for analyses, yielding a sample of 418 youths. Mean age at baseline was 9.53 years (*SD* = 0.51, range: 8–11) and mean age at Y_2_ was 11.69 years (*SD* = 0.72, range: 9–13). Additional demographic information can be found in Table 1. Figure 3 depicts a flow diagram of youth from the full ABCD dataset to the sample used in the present study.

**Table 1:** Demographic and clinical characteristics.

	NSSI *n* = 209	Control *n* = 209	test	*p* (2-tailed)	Effect size
Demographic					
Age (*m* [*SD*])	9.5 (0.52)	9.55 (0.51)	*t*(416) = 0.97	0.334	*d* = -0.09
Sex (*n*; M/F)	72/137	72/137	χ^2^(1) = 0.00	1.00	ϕ=0.00
Gender (*n*; M/F/O)	72/136/1	72/136/1	χ^2^(2) = 0.00	1.00	*V* = 0.00
White (*n*)	178/31	164/45	χ^2^(1) = 3.15	0.076	ϕ = 0.09
Black (*n*)	41/168	42/167	χ^2^(1) = 0.02	0.902	ϕ = −0.01
Native American (*n*)	11/198	<10/201	χ^2^(1) = 0.50	0.481	ϕ = 0.03
Pacific Islander^a^ (*n*)	<10/207	<10/207	χ^2^(1) = 0.00	1.00	ϕ = 0.00^d^
Asian^b^ (*n*)	10/199	<10/206	χ^2^(1) = 3.89	**0.049**	ϕ = 0.09^d^
Other/unknown race^c^ (*n*)	11/198	<10/201	χ^2^(1) = 0.50	0.481	ϕ = 0.03
Hispanic/Latino (*n*)	36/173	35/174	χ^2^(1) = 0.02	0.896	ϕ = 0.01
Parents married (*n*)	141/68	145/64	χ^2^(1) = 0.18	0.674	ϕ = −0.02
MRI manufacturer (*n*; GE/Siemens)	51/158	45/164	χ^2^(1) = 0.49	0.485	ϕ = 0.03
UPPS-P Scale (*m* [*SD*])					
Negative urgency	8.74 (2.65)	8.33 (2.68)	*t*(416) = −1.56	0.120	*d* = 0.15
Lack of premeditation	7.91 (2.46)	7.71 (2.36)	*t*(416) = −0.83	0.405	*d* = 0.08
Sensation seeking	10.09 (2.76)	9.63 (2.73)	*t*(416) = −1.71	0.088	*d* = 0.17
Positive urgency	8.16 (2.98)	7.84 (3.08)	*t*(416) = −1.10	0.273	*d* = 0.11
Lack of perseverance	7.48 (2.41)	7.01 (2.03)	*t*(416)-2.20	**0.029**	*d* = 0.22
GFA (m [SD])					
Left cingulum	0.096 (0.006)	0.096 (0.005)	*t*(416) = 1.16	0.246	*d* = −0.11
Left cingulum frontal parahippocampal	0.106 (0.007)	0.107 (0.007)	*t*(416) = 1.88	0.061	*d* = −0.18
Left cingulum frontal parietal	0.102 (0.006)	0.103 (0.006)	*t*(416) = 2.04	**0.042**	*d* = −0.20
Left cingulum superior longitudinal fasciculus	0.085 (0.006)	0.085 (0.005)	*t*(416) = 0.23	0.818	*d* = −0.02
Left cingulum parahippocampal parietal	0.086 (0.006)	0.087 (0.005)	*t*(416) = *1.00*	0.317	*d* = −0.10
Left cingulum parahippocampal	0.084 (0.006)	0.085 (0.006)	*t*(416) = 0.86	0.389	*d* = −0.08
Left cingulum parolfactory	0.088 (0.007)	0.089 (0.007)	*t*(416) = 1.92	0.056	*d* = *−*0.19
Left uncinate fasciculus	0.087 (0.005)	0.088 (0.005)	*t*(416) = 0.64	0.521	*d* = *−*0.06
Right cingulum	0.093 (0.005)	0.094 (0.005)	*t*(416) = 0.93	0.355	*d* = *−*0.09
Right cingulum frontal parahippocampal	0.100 (0.007)	0.101 (0.007)	*t*(413) = 2.24	**0.026**	*d* = *−*0.22
Right cingulum frontal parietal	0.097 (0.006)	0.098 (0.006)	*t*(416) = 1.93	0.055	*d* = −0.19
Right superior longitudinal fasciculus	0.086 (0.005)	0.087 (0.005)	*t*(416) = 1.29	0.198	*d* = −0.13
Right cingulum parahippocampal parietal	0.087 (0.006)	0.086 (0.006)	*t*(416) = −1.24	0.215	*d* = 0.12
Right cingulum parahippocampal	0.085 (0.006)	0.084 (0.006)	*t*(416) = −1.21	0.228	*d* = 0.12
Right cingulum parolfactory	0.093 (0.008)	0.094 (0.007)	*t*(415) = 0.95	0.342	*d* = *−*0.09
Right uncinate fasciculus	0.089 (0.005)	0.089 (0.005)	*T*(416) = 0.87	0.384	*d* = *-*0.09

Bold values indicate statistically significance *p* < 0.05.

Statistical tests are presented for descriptive purposes.

*m [SD]* Mean [Standard Deviation], *M* male, *F* female, *O* other.

^a^Pacific Islander composite combining Samoan and Other Pacific Islander.

^b^Asian composite combining Asian Indian, Chinese, Filipino, Japanese, Korean and Other Asian.

^c^Other/Unknown composite combining *Other race* and *Unknown race*.

^d^Fisher’s Exact Test was used where indicated for accuracy of testing in smaller samples.

### Logistic regression model results

Four participants (three female and one male) were dropped from LASSO analyses due to missing GFA values for at least one tract. The LASSO logistic regression with site-grouped cross-validation selected 16 predictors from the full set of main effects and sex **×** predictor interactions. The best lambda was 0.011 with a mean cross-validation AUC of 0.6037 and 95% confidence interval (CI) of 0.542–0.666. Selected main effects included the right frontal-parahippocampal and parahippocampal-parietal sections of the cingulum, right and left superior longitudinal fasciculus and parahipocampal sections of the cingulum, left frontal-parietal parolfactory section of the cingulum, negative urgency, and intracranial volume. Selected sex × predictor interaction terms included the right superior longitudinal fasciculus and left parolfatory sections of the cingulum, the right uncinate fasciculus, lack of perseverance, negative urgency, and sensation seeking. Selected predictors had moderate to good stability across folds, with right superior longitudinal fasciculus GFA and sex × right superior longitudinal fasciculus GFA having the lowest stability at 50% and 62.5%, respectively, and other selected predictors being present in 75% or more of folds. Selected predictors were entered as fixed effects into a mixed-effects logistic regression with a random intercept for site. There was negligible between-site variability after accounting for fixed effects as the site-level random effect variance estimate was zero. Results for all selected predictors can be found in Table 2.

**Table 2:** Main effects and interactions from mixed-effects logistic regression.

Fixed effects main effects						
*Predictor*	*β*	*SE*	*OR*	*95% CI*	*z*	*p*
Intercept	0.015	0.108	1.015	[0.822, 1.253]	0.138	0.890
Right cingulum frontal-parahippocampal GFA	−0.305	0.167	0.737	[0.533, 1.019]	−1.829	0.067
Left cingulum frontal-parietal GFA	−0.171	0.200	0.843	[0.570, 1.247]	−0.851	0.395
Left cingulum SLF GFA	0.299	0.160	1.348	[0.988, 1.840]	1.868	0.062
Right cingulum SLF GFA	−0.075	0.239	0.928	[0.581, 1.482]	−0.315	0.752
Right cingulum parahippocampal-parietal GFA	0.091	0.418	1.095	[0.483, 2.483]	0.217	0.828
Left cingulum parahippocampal GFA	−0.323	0.194	0.724	[0.494, 1.061]	−1.663	0.096
Right cingulum parahippocampal GFA	0.607	0.456	1.836	[0.751, 4.488]	1.332	0.183
**Left cingulum parolfactory GFA**	**−0.458**	**0.219**	**0.633**	**[0.411, 0.974]**	**−2.088**	0.037*
**Negative Urgency**	**0.432**	**0.185**	**1.540**	**[1.072, 2.213]**	**2.333**	0.020*
Intracranial volume	−0.196	0.114	0.822	[0.658, 1.027]	−1.728	0.084

Sex × predictor interaction effects						
*Predictor*	*β*	*SE*	*OR*	*95% CI*	*z*	*p*
Female × right cingulum SLF GFA	−0.112	0.262	0.894	[0.534, 1.495]	−0.427	0.669
**Female × left cingulum parolfactory GFA**	**0.560**	**0.248**	**1.750**	**[1.078, 2.840]**	**2.257**	0.024*
Female × right uncinate fasciculus GFA	−0.286	0.184	0.751	[0.524, 1.077]	−1.558	0.119
**Female × Lack of Perseverance**	**0.381**	**0.133**	**1.463**	**[1.126, 1.901]**	**2.852**	0.004**
**Female × Negative Urgency**	**−0.513**	**0.232**	**0.598**	**[0.380, 0.942]**	**−2.213**	0.027*
**Female × Sensation Seeking**	**0.305**	**0.137**	**1.357**	**[1.039, 1.772]**	**2.230**	0.026*

Bold values indicate statistically significance *p* < 0.05.

Interaction terms reflect the difference in the predictor’s association with outcome in females relative to males. Confidence intervals computed as exp(β ± 1.96 × SE).

*OR* odds ratio, *SLF* superior longitudinal fasciculus, *GFA* generalized fractional anisotropy.

*p < 0.05; **p < 0.01.

There were two significant main effects: lower GFA in the left parolfactory segment of the cingulum (*β* = −0.46, *SE* = 0.22, *z* = −2.09, *p* = 0.037, OR = 0.63, 95% CI [0.41–0.97]) and higher negative urgency (*β* = 0.43, *SE* = 0.19, *z* = 2.33, *p* = 0.020, OR = 1.54, 95% CI [1.07–2.21]) was associated with increased likelihood of Y2 NSSI. Several sex **×** predictor interactions were significant. GFA of the left parolfactory segment of the cingulum showed a weaker or reversed relation with Y2 NSSI in females relative to males (*β* = 0.56, *SE* = 0.25, *z* = 2.26, *p* = 0.024, OR = 1.75, 95% CI [1.08–2.84]). Similarly, the relation between negative urgency and Y2 NSSI was weaker in females relative to males (*β* = −0.51, *SE* = 0.23, *z* = −2.21, *p* = 0.027, OR = 0.60, 95% CI [0.38–0.94]). Conversely, sensation seeking (*β* = 0.31, *SE* = 0.14, *z* = 2.23, *p* = 0.026, OR = 1.36, 95% CI [1.04–1.77]) and lack of perseverance (*β* = 0.38, *SE* = 0.13, *z* = 2.85, *p* = 0.004, OR = 1.46, 95% CI [1.13–1.90]) were positively associated with Y2 NSSI in females.

## Discussion

This is the largest study to date investigating white matter microstructure associated with NSSI, the first to our knowledge to evaluate white matter and impulsivity prospectively, and the first to examine sex differences. We focused specifically on white matter tracts previously implicated in studies of NSSI that also serve to connect frontolimbic regions [26, 27]. By examining differences between males and females, different patterns emerged, highlighting the importance of considering sex when examining the neurobiology associated with NSSI. Significant main effects indicated that lower GFA of the left parolfactory segment of the cingulum and higher negative urgency was associated with increased likelihood of Y2 NSSI. We found significant sex **×** predictor interactions in which females demonstrated that higher lack of perseverance and sensation seeking at baseline were associated with increased likelihood of NSSI at Y_2._ In males, lower GFA of the left parolfactory section of the cingulum and higher negative urgency at baseline were associated with increased likelihood of NSSI at Y_2_.

While the main effect of lower GFA of the parolfactory section of the cingulum being associated with NSSI onset was consistent with hypotheses, there was also a significant sex interaction. Specifically, this relation was driven by males, with females showing an attenuated if not opposing pattern. The parolfactory section is anterior and dorsal, serving the anterior cingulate cortex and extending through the mid-cingulate into the posterior cingulate [49]. These regions are involved in emotion processing, learning, self-reflection, and decision-making [50]. Prior work has indicated that NSSI is associated with lower white matter organization relative to non-clinical controls [25–28]. Whereas higher white matter organization has been associated with cognitive control deficits in non-clinical controls, but not in adults with depression [51]. Potential explanations for these contrasting findings among females may be due to age of the present sample and the examination of white matter prior to NSSI onset. Interestingly, overall GFA of the left cingulum was associated with longer duration of NSSI in a prior study of females [26], suggesting that lower GFA may occur following NSSI onset in females, or at least later in adolescent development. This underscores the importance of examining the temporal relation between GFA and NSSI as more data are released from the ABCD sample (i.e., data collected later when the sample has reached a later stage of adolescence).

We also found higher lack of perseverance and sensation seeking in females was associated with increased likelihood of NSSI at Y_2_. Lack of perseverance involves difficulty following through with tasks and is associated with low conscientiousness [52, 53]. Individuals with NSSI show significantly higher lack of perseverance relative to non-clinical controls, which was positively correlated with emotion regulation difficulties and lifetime frequency and maintenance of NSSI [54–56]. Interestingly, higher lack of perseverance has been associated with using fewer methods of NSSI, which is contrary to expectations as more NSSI methods is typically associated with greater severity [54, 57]. However, lack of perseverance in the context of NSSI may be associated with reluctance to engage in alternative coping strategies that are more adaptive, leading to greater avoidance and build-up of internalized negative emotions, which eventually manifest as emotion dysregulation and NSSI. This difficulty in engaging in alternative strategies may also extend to the use of different NSSI methods [56]. Longitudinal studies that more comprehensively evaluate NSSI (including frequency, methods and other characteristics) are needed to evaluate this relationship. Our finding of higher sensation seeking being associated with NSSI onset in females aligns with prior research [58]. Interestingly, lower sensation seeking is associated with NSSI in adults, with females having lower sensation seeking relative to males [59]. As the latter study examined young adults as opposed to youths, our finding highlights the importance of considering the developmental course of these constructs in understanding their association with NSSI. Higher levels of sensation seeking may predispose youth to initiate NSSI while this relation may weaken as NSSI becomes more entrenched. This would suggest that higher sensation seeking would be associated with a younger age of onset, which was not found in prior research [58]. Nonetheless, this previous study was limited by retrospective reporting in addition to sensation seeking measured after NSSI onset.

Our hypotheses of lower GFA and higher negative urgency being prospectively associated with NSSI was supported for males. Overall, males had significantly higher scores on negative urgency relative to females, and iNSSI males had significantly higher negative urgency relative to male controls. Considering that the iNSSI group was carefully matched by both demographic and clinical characteristics, it is possible that negative urgency is more specific to NSSI risk in males, whereas it is linked to psychopathology more generally in females.

Limitations of this study include the lack of a comprehensive assessment of NSSI, such as frequency, intensity, methods, and functions. In particular, the Y_2_ KSADS assessed current and lifetime experiences, as opposed to experiences since last assessment. Given this, it is possible that some youth with NSSI at Y_0_ may have initially denied this behavior at Y_0_ but later endorsed this positively at Y_2_. Future research is needed to continue examining how these relations manifest over time as the present study only examines NSSI onset at Y_2_ in the ABCD Study, which is a relatively early age of onset. Another limitation is that given the observational study design that examined associations between variables, causal inferences cannot be made. Further, the two-stage analytic approach (LASSO followed by logistic regression) should be interpreted cautiously as results do not account for uncertainty in variable selection. Finally, while GFA provides a diffusion metric less susceptible to influences such as crossing fibers and complexity of white matter tracts, it is still limited in its ability to definitively reflect the health of white matter tracts. Strengths of this study include its prospective design, sample size, and carefully matched control group that allows for examining predictors that are more specific to NSSI as opposed to general psychopathology. Further, the sample size allowed for examining the sexes separately, which is highly valuable given the association between NSSI and males is underexplored.

Overall, the present study demonstrates that aberrant white matter microstructure and specific facets of impulsivity are prospectively associated with onset of NSSI in youths. Importantly, these relations differ depending on sex. This work provides an important foundation for future longitudinal research designed to investigate and validate key neural, behavioral, and personality characteristics that may pave the way for improved prevention and intervention strategies. Such strategies may include identification of risk factors and proactively helping children to develop adaptive coping strategies, which may support healthy neurodevelopment and reduce likelihood of future NSSI.

## Data Availability

The ABCD data is publicly available to researchers upon receipt of a Data Use Agreement.
